# Anti-Herpes Simplex Virus Efficacy of Silk Cocoon, Silkworm Pupa and Non-Sericin Extracts

**DOI:** 10.3390/antibiotics10121553

**Published:** 2021-12-19

**Authors:** Kanyaluck Jantakee, Panchika Prangkio, Aussara Panya, Yingmanee Tragoolpua

**Affiliations:** 1 Department of Biology, Faculty of Science, Chiang Mai University, Chiang Mai 50200, Thailand; kanyaluckjan@gmail.com; 2 Department of Chemistry, Faculty of Science, Chiang Mai University, Chiang Mai 50200, Thailand; panchika.p@cmu.ac.th; 3 Research Center in Bioresources for Agriculture, Industry, and Medicine, Faculty of Science, Chiang Mai University, Chiang Mai 50200, Thailand

**Keywords:** antiviral activity, epithelial cell, herpes simplex virus, non-sericin, silk cocoon, silkworm pupa

## Abstract

Herpes simplex virus (HSV) infections are prevalent worldwide and are the cause of life- threatening diseases. Standard treatment with antiviral drugs, such as acyclovir, could prevent serious complications; however, resistance has been reported specifically among immunocompromised patients. Therefore, the development of an alternative approach is needed. The silk cocoon derived from silkworm, *Bombyx mori,* has been recognized for its broad-spectrum biological activity, including antiviral activity; however, its effects against HSV infection are unknown. In this study, we investigated the inhibitory effects of silk extracts derived from the cocoon shell, silk cocoon, silkworm pupa and non-sericin extract, on blocking HSV-1 and HSV-2 binding to host cells, resulting in the inhibition of the virus infection in Vero cells. Non-sericin extract demonstrated the greatest effectiveness on inhibiting HSV-1 and HSV-2 binding activity. Moreover, the virucidal effect to inactivate HSV-1 and HSV-2 was determined and revealed that non-sericin extract also exerted the highest potential activity. Using the treatment of non-sericin extract in HSV-2-infected HeLa cells could significantly lower the HSV-induced cell death and prevent inflammation via lowering the inflammatory cytokine gene expression. The non-sericin extract was analyzed for its bioactive compounds in which gallic acid, flavonoid and xanthophyll were identified, and might have partially contributed to its antiviral activity. The finding in our study suggested the potential of silk extract as an alternative therapeutic treatment for HSV infection.

## 1. Introduction

Herpes simplex virus (HSV) infection is a common public health problem worldwide, which affects approximately 60–95% of the adult population [1]. The infection causes recurrent vesiculoulcerative lesions and may lead to life-threatening conditions. HSV is a member of the family *Herpesviridae,* consisting of two serotypes, including herpes simplex virus type 1 (HSV-1) and herpes simplex virus type 2 (HSV-2). The viral particle contains an icosahedral capsid that is surrounded by amorphous tegument proteins and a lipid envelope [2]. The genomic material of the virus is a linear double strand DNA, approximately 150 kbp in size (152 kbp for HSV-1 and 155 kbp for HSV-2). The replication cycle of HSV begins with the interaction of the virus and the host cell receptor, which triggers the viral entry by fusing the viral envelope with the host cell membranes. Subsequently, the nucleocapsid and tegument proteins are internalized into the cytoplasm where the replication and transcription processes of HSV α, β and γ genes or proteins occur. The new virions are assembled in nucleus, released enveloped virus and spread into other cells [3].

Both types of HSV spread mainly through close contact from person to person via infected secretions. HSV-1 infection typically causes orofacial mucocutaneous lesions and HSV-2 is mainly associated with genital herpes. The infection of HSV can cause primary and recurrent infections upon stimulation by means of health hazards, [4] and might lead to chronic skin disease and lesions at any site on the human body [5,6,7]. Disease severity depends on the mode of transmission, age and immunocompetency of the host [7]. Prodromal symptoms may include multiple round ulcers or superficial erosions which are commonly followed by classic vesiculoulcerative lesions. On the other hand, the infection in children may cause serious symptoms such as erythema and swelling of the gingiva, drooling, foul smelling breath and anorexia [5,8], and may lead to substantial psychological morbidity [2]. Importantly, HSV-2 infection has been reported as a cofactor associated with human cervical cancer carcinogenesis [9,10].

Acyclovir is a primary drug for herpesvirus, which targets the DNA replication pathway [11]. Long-term prophylaxis and treatment has led to the development of drug resistance especially in the immunocompromised patients. New antiviral strategies have been developed for the treatment of HSV infections, targeting viral attachment, viral egress, virion maturation and immunomodulation of the host cell. These approaches could be an effective strategy for controlling symptoms of herpesvirus infections and decrease the development of traditional drug resistances as well [12,13]. However, novel antiviral drug could not replace the use of conventional drugs, and the study of the new drugs are still ongoing. Furthermore, the infection of HSV is still a risk factor of carcinogenesis, caused by damage of the epithelial cells with the induction of local chronic inflammation. Therefore, it is worthwhile to explore novel bioactive agents from natural sources as an alternative prophylaxis and treatment of which the actions are different from the synthetic drugs.

Silkworm, *Bombyx mori,* is an economically important insect, and its natural silk product has been used in the textile industry for decades. Apart from the industrial utility, silkworm is a functional food with a high content of proteins and nutrients. The silkworm pupa has also been reported to be a good source of nutrients. [14]. The cocoon is generally composed of 70% fibroin, 20–30% sericin and 1–2% several chemical compositions, such as carbohydrates, lipids and pigments [15]. The silk contains mainly two proteins, including sericin and fibroin, which are commonly used as degradable biomaterials in biomedical fields [15,16]. Interestingly, the silk proteins exerted several pharmacological properties, including the induction of cell proliferation, antioxidant anti-tyrosinase, anti-inflammation, anti-cancer and antibacterial activities [17]. These properties were mainly found in the sericin protein [17]. Moreover, bioactive substances are also isolated from many parts of the silk cocoon, for example, flavonoids and carotenoids are isolated from the shell of Thai silk, which showed antioxidant activity [18]. Although several phrenological activities of bioactive substances of silkworm components, such as sericin protein and non-sericin have been reported, the antiviral properties have not been previously investigated. In our study, we demonstrated the anti-viral activity of silkworm components from inhibiting HSV infection. The non-sericin extracts showed inhibitory effects which significantly reduced HSV titer in Vero cells, and its inhibition had a direct effect on the viral particle during HSV infection. Furthermore, the antiviral activity of non-sericin was tested on human cells (HeLa) and revealed its potential for lowering the virus infection rate and preventing inflammation and virus-induced cell death.

## 2. Results

### 2.1. The Inhibitory Effect of Silk Cocoon and Silkworm Pupa Extracts against HSV-1 and HSV-2 Infection via Blocking Binding Activity in Vero Cell

**Figure 1:**
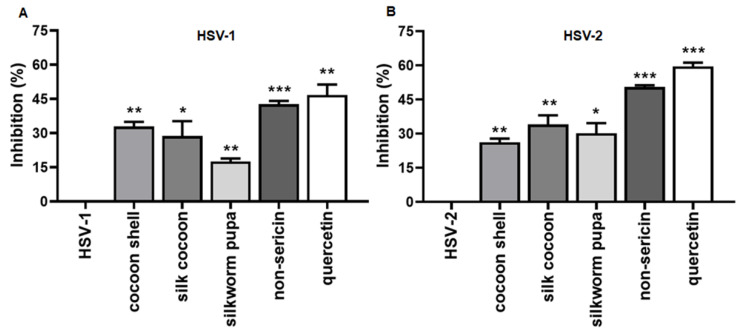
Effect of the extracts on HSV binding activity to the Vero cells. The extracts were treated into the cells at the same time with HSV-1 or HSV-2. The treated cells were incubated at 4 °C for 3 h to allow the virus to bind to the host cells. Extracts of cocoon shell, silk cocoon, silkworm pupa, non-sericin and quercetin were used at concentrations of 2.5 mg/mL, 1.25 mg/mL, 0.625 mg/mL, 0.625 mg/mL and 80 µg/mL in the study, respectively. The reduction of plaque formation was measured and represented as percentage of inhibition of HSV-1 (**A**) and HSV-2 (**B**) binding activity (* indicates *p* < 0.05; ** indicates *p* < 0.01 and *** indicates *p* < 0.001).

The cytotoxicity of silk cocoon and silkworm pupa extracts was investigated in Vero cells and only the non-toxic doses were used in antiviral activity assay (Figure S1A). Trials to determine the antiviral effects of silk cocoon and silkworm pupa extracts on inhibiting HSV-1 and HSV-2 binding activity to the host cells were conducted in Vero cells. The virus, along with maximum tested concentrations of cocoon shell, silk cocoon, silkworm pupa or non-sericin extract were treated with the cells at the same time. In this study, cocoon shell, silk cocoon, silkworm pupa, non-sericin extracts and quercetin were used at concentrations of 2.5, 1.25, 0.625, 0.625 mg/mL and 80 µg/mL, respectively. The treated cells were incubated at 4 °C for 3 h to allow viral binding. The results indicated that the non-sericin extract treatment was the most effective on inhibiting HSV-1 and HSV-2 binding activity, exhibiting 42.71 ± 1.47 and 50.00 ± 0.00% of inhibition, respectively (Figure 1A or Figure 1B). Treatments with other types of silk cocoon extract also had inhibitory binding effects, but less than that observed in non-sericin extract. The inhibitory activity of cocoon shell, silk cocoon and silkworm pupa in either HSV-1 or HSV-2-infected cells was less than 30%. Meanwhile, the remarkable antiviral biding activity was also noted for quercetin with inhibition of 46.76 ± 4.58% of HSV-1 and 59.52 ± 1.68% of HSV-2. Moreover, the vehicles did not have any effect on the virus. Thus, the inhibitory effects against HSV binding were from the extracts.

**Figure 2:**
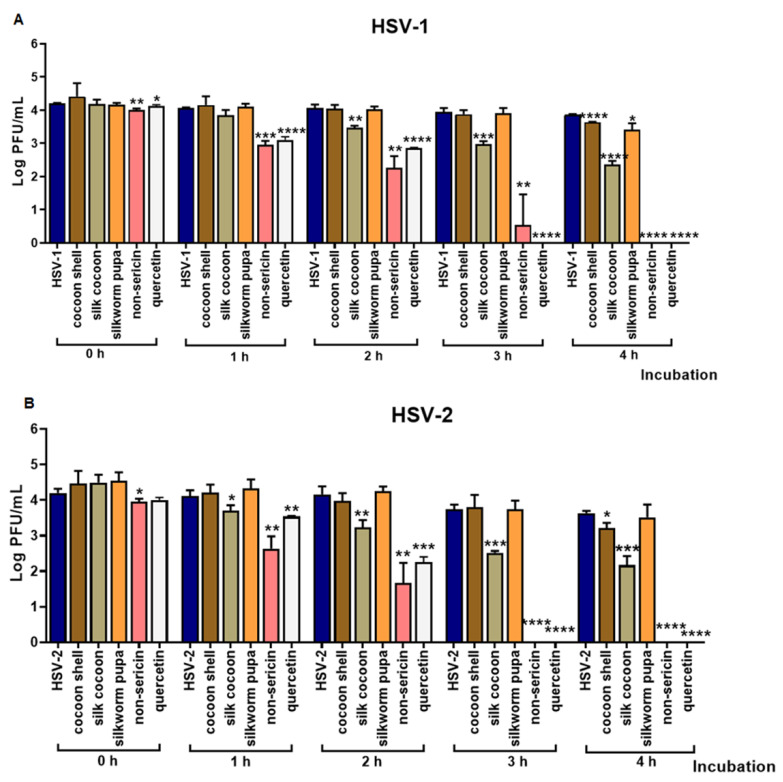
Direct effect of extracts on inactivation of HSV-1 and HSV-2 particles. The viral particles were incubated with the indicated concentrations of each extract at room temperature for 0, 1, 2, 3 and 4 h. Extracts of cocoon shell, silk cocoon, silkworm pupa, non-sericin and quercetin were used at concentrations of 2.5, 1.25, 1.25, 0.625 mg/mL and 80 µg/mL respectively. The infection capacity of HSV-1 (**A**) and HSV-2 (**B**) was evaluated by virus titration assay. The reduction of virus titer after treatment was represented as a log PFU/mL (* indicates *p* < 0.05; ** indicates *p* < 0.01; *** indicates *p* < 0.001, and **** indicates *p* < 0.0001).

We further investigated the direct effect of the extracts on HSV virus particles using kinetic inactivation assay. The HSV particles were incubated with extracts for 1, 2, 3 and 4 h. Cocoon shell, silk cocoon, silkworm pupa, non-sericin extracts and quercetin were used at concentrations of 2.5, 1.25, 1.25, 0.625 mg/mL and 80 µg/mL, respectively. Anti-HSV activity of vehicles was also investigated against HSV particles at the same condition and the vehicles did not show any effect to the virus. The virus titer was evaluated by plaque titration assay and represented in a log of virus titer (PFU/mL). The result indicated that treatment with 0.625 mg/mL of non-sericin extract was the most effective method to significantly reduce the amount of infectious viral particle. Non-sericin treatment completely inactivated HSV-1 and HSV-2 after 3 and 4 h of treatment, respectively (Figure 2A,B). Moreover, the quercetin compound had strong virucidal effect on both HSV-1 and HSV-2 after 3 h of treatment. The effects of cocoon shell, silk cocoon or silkworm pupa extracts were observed, and their inhibitory activity increased in a timely manner. These results demonstrated that the virucidal effects of the extracts directly inhibited HSV-1 and HSV-2 infection.

### 2.2. Non-Sericin Extract Reduced HSV-2 Infection in HeLa Cell

**Figure 3:**
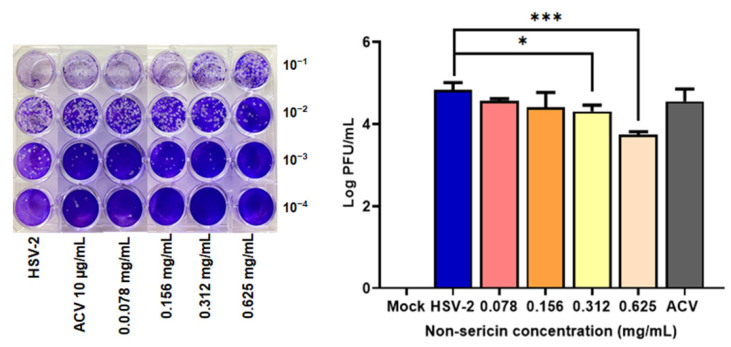
The inhibitory effect of non-sericin extract on infection in HeLa cells. The cells were treated with non-sericin extract during the virus attachment steps. After 48 h of infection, the viral titers in the culture supernatant were determined by plaque titration assay (* indicates *p* < 0.05 *** indicates *p* < 0.001).

To evaluate the efficiency of non-sericin extract as anti-herpes simplex virus agents, the antiviral property with non-sericin extract was determined in human HeLa cells. The effects on the reduction of HSV-2 virus production were investigated. The non-toxic concentrations of non-sericin extract were determined (Figure S1B) and used to establish the inhibitory activity during the virus attachment steps. The amounts of virus in culture supernatant were measured by using virus titration assay. Treatment of non-sericin extract at the concentration of 0.078–0.625 mg/mL greatly decreased the virus production in a dose dependent manner. Treatment with 0.625 mg/mL of non-sericin extract remarkably reduced the titer of HSV-2 with approximately 10 times less than the untreated cells (Figure 3).

### 2.3. Non-Sericin Extract Protected HSV-2-Induced Cell Death and Inflammation in HeLa Cells

**Figure 4:**
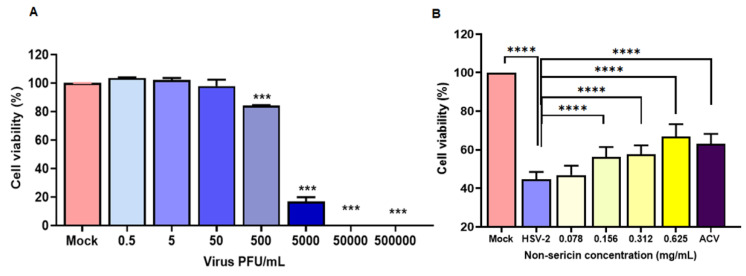
Effect of HSV infection on host cell viability. The cell viability of HeLa after HSV-2 infection (**A**) and the effect of non-sericin treatment to rescue HeLa cell viability were determined (**B**) by using cell viability assay (*** indicates *p* < 0.001, and **** indicates *p* < 0.0001).

To address the consequence of HSV-2 infection in HeLa cells, the cell viability was determined after HSV-2 infection. The effects of cell viability on HeLa cells were further examined. The HSV-2 at approximately 0.5 – 5 × 10^5^ PFU/mL was infected into HeLa cells. The result showed that increasing the amount of virus showed a significant effect on cell viability in a dose dependent manner (Figure 4A). We therefore investigated whether the treatment of non-sericin extract would protect the cells from HSV-2-induced cell death. HeLa cells were infected with 5 × 10^3^ PFU/mL of HSV-2 and treated together with non-sericin extract at a concentration of 0.078–0.625 mg/mL. Interestingly, non-sericin extract exhibited a significant increase in cell viability during HSV-2 infection (Figure 4B).

**Figure 5:**
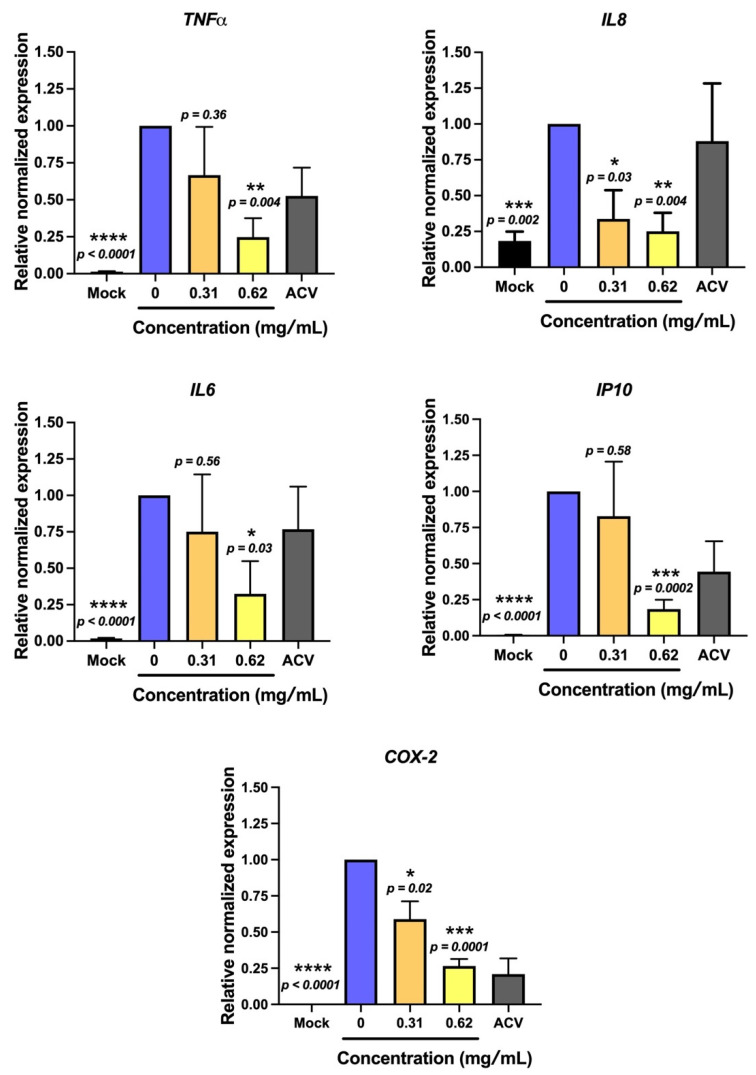
Effect of non-sericin extract on lowering inflammatory gene expression in HSV-2 infected cells. HeLa was infected with HSV-2 in the absence or presence of non-sericin extract at the concentrations of 0.31 and 0.62 mg/mL and measured for the gene expression level of *TNFα*, *IL6*, *IL8*, *IP10* and *COX-2* by real-time PCR after 48 h after infection. The normalized gene expression of *TNFα*, *IL6*, *IL8*, *IP10* and *COX-2* were represented relative to non-treatment control (set as 1). The statistical differences were analyzed comparing to the expression of the non-treatment control (* indicates *p* < 0.05, ** indicates *p* < 0.005, *** indicates *p* < 0.0005 **** indicates *p* < 0.0001).

Furthermore, the responses of HSV-2 infection on the modulating host were also investigated. The alteration of the inflammatory cytokine gene expression, which plays a role on inflammation during the infection, was evaluated, including interleukin (*IL)6*, *IL8*, tumor necrosis factor (*TNF)α* and *IP10*. HSV-2 infection remarkably upregulated the transcript levels of *TNFα, IL6, IL8* and *IP10* genes. Interestingly, treatment with non-sericin concentration showed the great potential to lower those inflammatory gene expressions (Figure 5). At the highest tested concentration (0.62 mg/mL), the treatment could significantly lower the expression of *TNFα, IL6, IL8* and *IP10* to 0.24, 0.25, 0.32 and 0.18 relative to non-treatment control (set as 1.0). Furthermore, we investigated the effect of non-sericin treatment on *COX-2* expression. COX-2 is well-known as a key mediator of inflammatory pathway. As expected, the result demonstrated that the expression of *COX-2* gene was significantly reduced in dose dependent manner after treatment with non-sericin extract (Figure 5), which emphasizes that non-sericin extract had the efficacy to reduce inflammation in infected cells.

### 2.4. Bioactive Compounds in Silk Cocoon Extracts

To identify the bioactive compounds in the extracts, the total amounts of flavonoids, phenolic compound and antioxidant levels of the silk cocoon and silkworm pupa extracts were evaluated using HPLC analysis, DPPH and ABTS assays. Our results indicated that cocoon shell, silk cocoon, silkworm pupa and non-sericin extracts contained high levels of flavonoid and phenolic compound. The antioxidant activities of the extracts also confirmed the bioactivity of flavonoid and phenolic compound contents (Table 1). Silkworm pupa extract showed the highest phenolic compound of 54.57 ± 3.16 mg gallic acid equivalents (GAE) per gram of extract. The highest antioxidant activities of 60.57 ± 5.81 mg GAE/g extract, and 275.06 ± 12.07 mg Trolox equivalent antioxidant capacity (TEAC) per gram extract were also observed from silkworm pupa extract using DPPH and ABTS methods. However, non-sericin extract showed the highest flavonoid compound by 6.33 ± 0.39 µg quercetin equivalents (QAE) per gram of extract.

**Figure 6:**
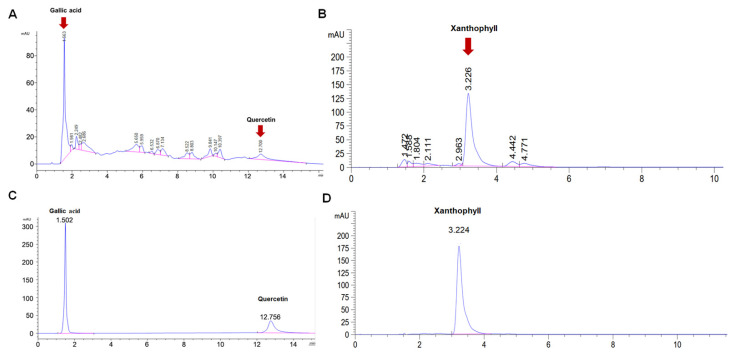
HPLC profile of non-sericin extract (**A**,**B**) compared to the standard gallic acid (**C**), quercetin (**C**), and xanthophyll (**D**) for analysis of bioactive compounds in non-sericin extract.

To determine the bioactive compound in non-sericin extracts, and to confer the most effective anti-viral activity, the HPLC analysis of the non-sericin extracts were performed using gallic acid, quercetin and xanthophyll as standard compounds for identification of bioactive compounds. The HPLC profiles showed that non-sericin extract contained gallic acid, quercetin and xanthophyll, which might be associated with anti HSV activity (Table 2, Figure 6).

## 3. Discussion

HSV infections cause diseases that are commonly found worldwide. Global prevalence of HSV-1 and HSV-2 infections vary remarkably by age, sex and geographical region. In 2016, HSV-1 and HSV-2 were reported to affect 66.6% and 13.2% of the world’s population, aged 0–49 years and 15–49 years, respectively [19]. Traditional herpes virus drugs have been developed to target the DNA replication pathway and essential viral proteins, such as the viral DNA polymerase. However, long-term prophylaxis and treatment can cause acquired resistance, especially in immunocompromised patients. Furthermore, the development of a prophylactic vaccination of HSV infection is challenging, due to the fact that the HSV infection develops a unique propensity to establish long-life latency, and currently, there is no approved available vaccine [20]. Therefore, it is important to search for novel pharmaceutical agents as a form of alternative therapy. The bioactive compound from natural sources was an attractive ingredient to be developed as a novel HSV therapeutic agent based on the broad spectrum of biological activity of natural products. In addition, natural agents are commonly safe and could lower the risk of the serious complications in long-term use. In this present study, we demonstrated the anti-HSV activity of the silk cocoon extract against HSV-1 and HSV-2 infections, which revealed the efficacy of silk cocoon extract to be developed as an alternative HSV drug.

Silk and related components have gained an increased interest for their biological properties and health benefits. Previously, natural bioactive agents of silk cocoon extracts, including silk sericin, silk fibroin, silkworm pupa and pigments of silk cocoon shell extracts, have been investigated for antibacterial activity, antioxidant activity, wound healing and anti-tumor effects [17,21,22,23]. In our experiment, the antiviral effects of silk cocoon extract, including cocoon shell, silk cocoon, silkworm pupa and non-sericin extract were demonstrated by blocking the HSV-1 and HSV-2 binding activity (Figure 1). Interestingly, non-sericin extract has shown the greatest effectiveness on inhibiting HSV-1 and HSV-2 binding activity. Previously, the direct interfering or competitive binding of viral particles to a specific cellular receptor has been reported to promote the inhibition at the initial stage of attachment and has lowered the chance of fusion steps of the viral envelope to host the cell membrane [24,25,26]. Our results suggest that the silk cocoon and silkworm extracts might interact with the surface of the host cell and interfere with HSV attachment to the specific receptor on the host cell [27,28], i.e., inhibition of viral glycoprotein C (gC) or glycoprotein B (gB) binding to heparan sulphate proteoglycan receptor [28]. The effects of natural compounds on disturbing the virus–host binding has been reported previously. The *Rubiaceae* extract has demostrated inhibitions to HSV-2 infection in pre-treatment conditions [29]. Other studies also revealed that natural substances such as polysaccharide extracts from *Eucheuma gelatinae*, chebulagic and chebulinic acids from *Terminalia chebula* interrupted the HSV viral attachment to the host cell [30,31].

To investigate the direct effect of the extracts on the virus, we further investigated the virucidal effect on HSV-1 and HSV-2. Moreover, the result of direct inactivation of the virus particle demonstrated that the non-sericin extract had the most virucidal effect against both HSV-1 and HSV-2 in a dose and time-dependent manner (Figure 2). We hypothesized that bioactive compounds in non-sericin extract might contribute to the inhibition through deconstructing the virion envelope structure or inducing the glycoprotein conformational change [32,33,34]. However, further investigation is needed to demonstrate the effect of extracts on viral structure i.e., cryo-EM to reveal changes of the virus surface.

HSV-2 is associated with coinfection with HPV or HIV, resulting in increasing the disease severity [35,36]. Furthermore, HSV-2 infection has been reported as a cofactor related to human cervical carcinogenesis [9,11]. Accordingly, we investigated the effect of the most potential extract, non-sericin extract, on inhibiting HSV-2 infection in human cervical adenocarcinoma HeLa cells. Treatment of non-sericin extract during the HSV-2 infection significantly reduced the virus production, which was concordant to the reduction of viral gene expression in infected cells (Figure 3). The effect of HSV-2 infection on the host cell viability and inflammation were further investigated to address the consequence of infection on the host cells, which might explain the involvement of HSV-2 chronic infection with disease severity and carcinogenesis. Infection of HSV-2 caused a significant reduction in HeLa cells and increased the gene expression of inflammatory cytokines *TNFα*, *IL6*, *IL8* and *IP10* (Figure 4 and Figure 5). Interestingly, treatment of non-sericin extract could protect the cells from HSV-2 infection and significantly prevent the HSV2-induced cell death. Likewise, it could lower the magnitude of *TNFα, IL6, IL8,* and *IP10* in a dose–dependent manner. Further studies are needed to clarify a decrease in inflammation infected cells after treatment with non-sericin extract by investigating the inflammatory enzyme of the *COX-2* gene, in which the enzyme is expressed in response to inflammatory process and has been found in several mechanism of disease. Interestingly, the result showed lower significantly expression of *COX-2* gene (Figure 5). The result suggested the potential of non-sericin extract to control the inflammation condition and might reduce the risk of chronic inflammation in HSV-2 infection.

The bioactive compounds in silk cocoon extracts were identified to explain the underlying biological activity. In general, silk cocoon is composed of 70% fibroin, 20–30% sericin and 1–2% non-protein material. [15,17]. Previously, several bioactive compounds from silk extracts have been reported for their activities against virus infections. Silk cocoon containing quercetin and other flavonoids had an inhibitory effect on HIV reverse transcriptase, HSV DNA polymerase beta, influenza and poliovirus replication [37,38]. The synthesized sulfated fibroin peptides showed anti-human immunodeficiency virus replication in vitro, which disturbed the adsorption of virus particles in CD4+ cells [39]. The silkworm extract contained natural iminosugars, such as 1-deoxynojirimycin (1-DNJ), and exhibited antiviral effects by inhibiting the glycosylation of the hepatitis viral envelope proteins [40]. Moreover, lipase from the digestive juice of *B. mori* larvae exposed strong antiviral activity against *B.mori* nucleopolyhedrovirus (BmNPV) [41]. We first identified the phenolic and flavonoid compounds, the most important compounds that can be found in natural substances and recorded the potential effects of their biological activity (Table 1). The results showed that all extracts used in this study contained the phenolic and flavonoid compounds, but the proportions were varied among the extracts. The proportion of the extract with high phenolic compounds exhibited the most potential antioxidant activity, judged by DPPH (mg GAE/g extract) and ABTS (mg TEAC/g extract) assays. This extract is mainly associated with antioxidant activity in silkworm pupa with the highest phenolic contents. Moreover, the phenolic and flavonoid compounds were likely to contribute to the antiviral effect in our study since these bioactive compounds were found in the non-sericin compartment.

Non-protein composition or the non-sericin compartment includes 0.4–0.8% wax matter, 1.2–1.6% carbohydrates, 0.7% inorganic matter and 0.2% pigment [42,43]. Generally, it contains natural pigments that are typically flavonoids and carotenoids, which also exert important roles in antioxidant and anti-tyrosinase activities [43,44,45]. However, the presence of additional chemical component has been reported in the non-sericin compartment as well. We further identified the reported chemical substance in our non-sericin extract by using the HPLC wherein the gallic acid, quercetin and xanthophyll compounds were used as standards. The HPLC results revealed that the chromatograms of the extracts consisted of three main peaks, which corresponded to the standard compounds (Figure 6). The result suggested that gallic acid, quercetin and xanthophyll might have contributed to the biological activity of non-sericin extract to inhibit HSV infection.

Therefore, the inhibition of HSV infection on Vero cells confirmed quercetin as one of the compounds in the crude extracts. However, we did not perform anti-HSV activity of gallic acid and xanthophyll in this study. The previous study also reported antiviral activity of flavonoid fraction; quercetin and kaempferol from *Ficus benjamina* leaf extract. These compounds showed the highest antiviral efficiency [46]. Moreover, quercetin isolated from Houttuynia cordata water extracts demonstrated anti-HSV activities, particularly on inhibition of viral binding, viral penetration, and host NF-κB activation [47]. Quercetin showed strong inhibition of HSV-1 infectivity on Raw 264.7 cells by suppression of viral entry and viral replication. Moreover, quercetin suppressed the expression of TLR-3, which led to the inhibition of inflammation [48]. Furthermore, quercetin and isoquercitrin demonstrated powerful antiviral activities against other human herpesviruses, including varicella-zoster virus and human cytomegalovirus [49].

Considering therapeutic use of the extract, the silk cocoon and silkworm pupa extracts have a mechanical pathway to inhibit the virus entry, which differs from the standard acyclovir (ACV) treatment that inhibited viral thymidine kinase (TK) and inhibits viral DNA polymerase [11]. Thus, it is possible for the extract to be used in a combination of treatment with ACV, or used as an alternative treatment, especially in ACV resistant patients. The natural substance has a lower toxicity, which may prevent serious complications. In addition, it provided a natural combination of various bioactive compounds, which theoretically is highly resistant to the virus escape mutation. The treatment of non-sericin components could lower not only the magnitude of virus infection, but also protect the HSV-induced cell death and prevent over-inflammation, suggesting the potential of the extract on a bidirectional approach to target the virus and reduce any harmful host responses. Findings in our study therefore support the use of silk extract as an alternative therapeutic treatment for HSV infection.

## 4. Materials and Methods

### 4.1. Silk Cocoon and Silkworm Pupa Extraction

The silk cocoons and shells were washed and cut into small pieces. The silk cocoons and shells were then boiled for 9 h in distilled water. Silkworm pupa were boiled for 1 h in distilled water. Additionally, a non-sericin fraction of silk cocoon shell was collected after macerating at room temperature for 24 h with 95% ethanol. Silk cocoon and pupae extracts were filtered through Whatman filter No. 1 and evaporated for solvent removal before being lyophilized to obtain dry extracts. The dried extracts of silk cocoon shell, silk cocoon and silkworm pupa were reconstituted with water while non-sericin extract was dissolved with dimethylsulfoxide (DMSO) (RCI Labscan, Bangkok, Thailand). Then, the reconstituted extract was aliquoted and stored at −20°C before investigation of anti-HSV activity. In addition, the quercetin compound was evaluated as a phytochemical control with valuable antiviral activity.

### 4.2. Cell Culture and Virus Propagation

The African green monkey kidney cells (Vero cells) and human cervical adenocarcinoma cell line (HeLa) were obtained from the division of Clinical Microbiology, Department of Medical Technology, Faculty of Associated Medical Sciences, Chiang Mai University. Vero cells and HeLa were cultured in Dulbecco′s Modified Eagle′s-Medium (DMEM) with 10% heat-inactivated fetal bovine serum (Gibco, Thermo Fisher Scientific, Waltham, MA, USA) and penicillin (100 Units/mL) and streptomycin (100 µg/mL) (Gibco, Thermo Fisher Scientific, Waltham, MA, USA) at 37°C in a humidified atmosphere of 5% CO_2_.

Standard strains of herpes simplex virus (HSV) type 1 strain F and type 2 strain G (HSV-1 and HSV-2) were propagated in Vero cells. The infected cells were maintained at 37 °C in a humidified atmosphere of 5% CO_2_ until they reached 80–90% cytopathic effect. Infected cells were frozen and thawed twice to collect the virus stock. The viruses were stored at −80°C and viral titer was measured by virus titration assay before used.

### 4.3. Anti-Herpes Simplex Virus Activity

#### 4.3.1. Cytotoxicity of Silk Extract on Vero Cell

The cytotoxicity of silk cocoon and pupa extracts was investigated by MTT assay. In brief, the Vero cells were seeded onto 96-well culture plates approximately 1.5 × 10^4^ cells/well. The two-fold dilutions of the extracts were treated with the cells and incubated for 72 h. Following, 3-[4,5-dimethylthiazol-2-yl]2,5-diphenyltetrazolium bromide (MTT) (Bio Basic, Markham, ON, Canada) was added and incubated for 4 h. The absorbance was measured at a wavelength of 540 nm with a reference wavelength of 630 nm and used to analyze the percentage of cell viability relative to the non-treated control, which was set a 100%. The half maximal cytotoxicity doses, or CD_50,_ was calculated from a dose–response curve.

#### 4.3.2. Viral-Host Cell Receptor Binding Activity Assays

The effect of silk cocoon and pupa extracts on viral binding activity to the host cell receptor was determined. In brief, the monolayers of Vero cells were pre-chilled at 4 °C for 1 h. HSV, 50 PFU/mL was then added to the combination with cold silk cocoon or silkworm pupa extracts. The treated cells were incubated for 3 h at 4 °C. Cells were then washed and incubated for 48 h. The plaque formation was observed and compared with the non-treated viral controls, and was used to calculate the percentage of HSV inhibition by using the following equation:

$$Inhibition\;\left(\%\right)=\left(\frac{Viral\;control-Treatment}{\mathrm{Viral}\;\mathrm{control}}\right)\times 100\;$$

#### 4.3.3. Direct Virus Inactivation Assay

The effects of silk cocoon and silkworm pupa extracts on direct inactivation of viral particles was determined. The extracts were mixed with HSV at approximately 1 × 10^4^ PFU/mL. Subsequently, the mixture was incubated for 1, 2, 3 and 4 h at room temperature. The infectious particles were measured after treatment by plaque titration assay. The virus titers of the residual virus were calculated and represented in logarithms of HSV (Log PFU/mL) and compared with the untreated virus control.

### 4.4. Antiviral Effect of Non-Sericin Extract in Human Cervical Adenocarcinoma Cell Line (Hela)

#### 4.4.1. Anti HSV-2 Efficacy of Non-Sericin Extracts in Hela Cells

The antiviral efficacy of non-sericin extracts was investigated in human target cells of the cervical adenocarcinoma HeLa cell line. In brief, HeLa cells (1.5 × 10^4^ cells/well) were cultured into a 96-well plate overnight. At the time of the experiment, HSV-2 (5 × 10^3^ PFU/mL) and the non-sericin extract were treated with HeLa cells. The cells were incubated for 1 h to allow for virus attachment, then the mixture was removed and replaced with fresh media. After 48 h of incubation, the supernatant was collected and the virus titration was conducted in Vero cells. The number of plaque formations was evaluated and compared with the viral controls.

#### 4.4.2. Effect of HSV Infection on HeLa Cell Viability and Protective Effect of Non-Sericin Extracts

The effect of HSV-2 infection on Hela cell viability was determined by using PrestoBlue cell viability reagent (Invitrogen Corporation, Carlsbad, CA, USA). In brief, HeLa cells (1.5 × 10^4^ cells/well) were cultured in a 96-well plate and infected with HSV-2 ranging from 0.5–5 × 10^5^ PFU/mL. After 48 h of incubation, PrestoBlue reagent was added for detection cell viability. The effect of non-sericin extract treatment on the protection of the cells from HSV-2-induced cell death was also determined. Approximately 5 × 10^3^ PFU/mL of HSV-2 were co-treated with the indicated concentrations of extract for 1 h. The mixture was then removed and replaced with media. The percentage of cell viability was determined relative to the non-treated control and was compared with the infected cell control.

#### 4.4.3. Effect of HSV Infection on Inducing Inflammation in HeLa and the Inhibitory Effect of Non-Sericin Extracts

The effect of HSV-2 infection on inducing inflammation in HeLa was investigated in real-time PCR. The HeLa cells were infected with HSV-2 in the presence or absence of non-sericin extract as described above. The cells were harvested 48 h after infection and isolated for total RNA using TRIzol (Invitrogen Corporation, Carlsbad, CA, USA). The cDNA synthesis was performed using Tetro cDNA Synthesis Kit (Meridian Bioscience, Memphis, TN, USA) and used as the template for real-time PCR using the primers specific to *TNF alpha*, *IL8*, *IL6*, *IP10* and *COX-2* (Table S1).

### 4.5. The Antioxidant Activity and Quantification of Total Phenolic Compounds of Silk Cocoon and Pupa Extracts

#### 4.5.1. Investigation of Antioxidant Activity of Silk Cocoon and Pupa Extracts

The 2,2-diphenyl-1-picrylhydrazyl (DPPH) and ABTS radical cation is a decolorization assay that is used to test the antioxidant activity of silk cocoon extracts. Silk cocoon and silkworm pupae extracts were prepared at different concentrations. The extracts were then mixed with 0.1 mM DPPH reagent or 7 mM ABTS+ cation radical reagent following absorbance was estimated at 517 and 734 nm, respectively. The antioxidant activity of DPPH assay was expressed as mg of gallic acid equivalents per gram of extract (mg GAE/g extracts). Moreover, the antioxidant activity of ABTS assay was expressed as mg of Trolox equivalents per gram of extract (mg TEAC/g extracts).

#### 4.5.2. Investigation of Total Phenolic Compound

The Folin–Ciocalteu method was used to determine the total phenolic content. Silk cocoon and silkworm pupa extracts were mixed with 50% Folin–Ciocalteu’s. The reaction was then measured at a wavelength of 725 nm. The total phenolic content was expressed as mg of gallic acid equivalents per gram of extract (mg GAE/g extracts).

#### 4.5.3. Investigation of Total Flavonoid Compound

The total flavonoid content was determined using the aluminium colorimetric method. The silk cocoon and silkworm pupa extracts were mixed with 10% aluminium chloride and 0.5 M potassium acetate. After the incubation, the absorbance of the reaction was measured at a wavelength of 415 nm. The total flavonoid content will be expressed as mg quercetin equivalents per gram of extract (mg QAE/g extracts).

#### 4.5.4. High-Performance Liquid Chromatography (HPLC)

The bioactive compound profiles in non-sericin extract were determined by high performance liquid chromatography (HPLC). The quercetin, gallic acid and xanthophyll were used as standard compounds (Sigma–Aldrich, Darmstadt, Germany). The non-sericin extract was filtered through a 0.45 µm microfilter and 20 µL of the filtrated extract was injected into the HPLC system (Agilent Technologies, Santa Clara, CA, USA). The detection of quercetin and gallic acid compounds used the ZORBAX Eclipse XDB-C18 column (4.6 × 150 mm, 5 µm (Agilent Technologies)) with the mobile phase, and was carried out with a linear gradient of water and methanol, at a flow rate of 1 mL/min for 30 min. A UV photodiode array detector (267 nm) was used for the evaluation.

The xanthophyll compounds were also investigated with a conventional C18 column with isocratic HPLC systems of methanol and acetonitrile (9:1 (*v/v*)), with a flow rate of 1.0 mL per minute and a running time of 15 min at 30 °C The absorption spectra was recorded at 450 nm. for the analysis.

### 4.6. Statistical Analysis

The data from at least three independent experiments were analyzed by using GraphPad Prism vision 8 (GraphPad Software, San Diego, CA, USA). The data values were expressed as mean ± SD and the statistical significance was determined using Student’s *t* test.

## Figures and Tables

**Table 1:** The antioxidant activity, total phenolic and flavonoid contents of silk cocoon extracts accessed by DPPH and ABTS methods.

Type of Extracts	Total Phenolic	Total Flavonoid	Antioxidant Activity	
	(mg GAE/g Extract)	(µg QAE/g Extract)	DPPH Method ABTS Method	
			(mg GAE/g Extract)	(mg TEAC/g Extract)
Cocoon Shell	8.18 ± 1.73	0.71 ± 002	ND	ND
Silk Cocoon	34.29 ± 2.03	0.57 ± 0.04	8.27 ± 0.90	40.45 ± 2.77
Silkworm Pupa	54.57 ± 3.16 *	2.02 ± 0.17	60.57 ± 5.81 *	275.06 ± 12.07 *
Non-Sericin	36.47 ± 5.57	6.33 ± 0.39 *	10.09 ± 0.81	46.01 ± 5.25

The data were expressed as mean ± SD., * Statistical different (*p* ≤ 0.05).

**Table 2:** The chemical profile of non-sericin extract accessed by HPLC. The gallic acid, quercetin and xanthophyll content were evaluated.

Chemical Profile of Non-Sericin Extract	
Total gallic acid compounds (mg GAE/g extract)	3.35 ± 0.55
Total quercetin compounds (mg QAE/g extract)	1.67 ± 0.15
Total xanthophyll compounds (µg Xanthophyll/g extract)	365.00 ± 13.54

## Data Availability

Data is contained in this manuscript or supplementary materials.

## Supplementary Materials

The following supporting information can be downloaded at: https://www.mdpi.com/article/10.3390/antibiotics10121553/s1, Figure S1: The 50% cytotoxic concentration (CC50) of extracts after treatment of Vero cells (A) and non-sericin extract treated HeLa cell, Table S1: List of primers used to amplify cDNA transcripts levels from HSV-2 infected and non-sericin treated HeLa cell.

## References

[1] Marchi S., Trombetta C.M., Gasparini R., Temperton N., Montomoli E. (2017). Epidemiology of herpes simplex virus type 1 and 2 in Italy: A seroprevalence study from 2000 to 2014. J. Prev. Med. Hyg..

[2] Davison A.J. (2010). Herpesvirus systematics. Vet. Microbiol..

[3] Ibáñez F.J., Farías M.A., Gonzalez-Troncoso M.P., Corrales N., Duarte L.F., Retamal-Díaz A., González P.A. (2018). Experimental dissection of the lytic replication cycles of herpes simplex viruses in vitro. Front Microbiol..

[4] Zaichick S.V., Bohannon K.P., Smith G.A. (2011). Peripheral tissues, alphaherpesviruses and the cytoskeleton in neuronal infections. Viruses.

[5] Chayavichitsilp P., Buckwalter J.V., Krakowski A.C., Friedlander S.F. (2009). Herpes Simplex. Pediatr. Rev..

[6] Sanders J.E., Garcia S.E. (2014). Pediatric Herpes Simplex Virus Infections: An Evidence-Based Approach to Treatment. Pediatr. Emerg. Med. Pract..

[7] Smith T.T., Whitley R.J. (2017). SECTION 8 Clinical Microbiology: Viruses. Infect. Dis..

[8] Lachmann R. (2003). Herpes simplex virus latency. Expert Rev. Mol. Med..

[9] Gupta R., Warren T., Wald A. (2007). Genital herpes. Lancet.

[10] Fatahzadeh M., Schwartz R.A. (2007). Human herpes simplex virus infections: Epidemiology, pathogenesis, symptomatology, diagnosis, and management. J. Am. Acad. Dermatol..

[11] Piret J., Boivin G. (2011). Resistance of herpes simplex viruses to nucleoside analogues: Mechanisms, prevalence, and management. Antimicrob. Agents Chemother..

[12] Babar M., Najam-us-Sahar S.Z., Ashraf M., Kazi A.G. (2013). Antiviral drug therapy exploiting medicinal plants. J. Antivir Antiretrovir..

[13] Wisskirchen K., Lucifora J., Michler T., Protzer U. (2014). New pharmacological strategies to fight enveloped viruses. Trends Pharmacol. Sci..

[14] Kumar D., Dev P., Kumar R.V., Kumar D., Kundapur R. (2015). Chapter 3 Biomedical Applications of Silkworm Pupae Proteins. Biomedical Applications of Natural Proteins.

[15] Zhang Y.Q. (2002). Applications of natural silk protein sericin in biomaterials. Biotechnol. Adv..

[16] Wongputtaraksa T., Ratanavaraporn J., Pichyangkura R., Damrongsakkul S. (2012). Surface modification of Thai silk fibroin scaffolds with gelatin and chitooligosaccharide for enhanced osteogenic differentiation of bone marrow-derived mesenchymal stem cells. J. Biomed. Mater. Res. Part B Appl. Biomater..

[17] Cao T.T., Zhang Y.Q. (2016). Processing and Characterization of Silk Sericin from *Bombyx mori* and Its Application in Biomaterials and Biomedicines. Mater. Sci. Eng..

[18] Prommuak C., De-Eknamkul W., Shotipruk A. (2008). Extraction of flavonoids and carotenoids from Thai silk waste and antioxidant activity of extracts. Sep. Purif. Technol..

[19] James C., Harfouche M., Welton N.J., Turner K.M., Abu-Raddad L.J., Gottlieb S.L., Looker K.J. (2020). Herpes simplex virus: Global infection prevalence and incidence estimates 2016. Bull. World Health Organ..

[20] Whitley R.J., Roizman B. (2001). Herpes Simplex Virus Infections. Lancet.

[21] Kaewkorn W., Limpeanchob N., Tiyaboonchai W., Pongcharoen S., Sutheerawattananonda M. (2012). Effects of Silk Sericin on the Proliferation and Apoptosis of Colon Cancer Cells. Biol. Res..

[22] Kunz R.I., Brancalhão R.M., Ribeiro L.D., Natali M.R. (2016). Silkworm sericin: Properties and biomedical applications. Biomed Res. Int..

[23] Saha J., Mondal I.H., Sheikh R.K., Habib A. (2019). Extraction, Structural and Functional Properties of Silk Sericin Biopolymer from *Bombyx mori* Silk Cocoon Waste. Int. J. Text. Sci..

[24] Harrison S.C. (2008). Viral membrane fusion. Nat. Struct. Mol. Biol..

[25] Schuksz M., Fuster M.M., Brown J.R., Crawford B.E., Ditto D.P., Lawrence R., Glass C., Wang L., Tor Y., Esko J.D. (2008). Surfen, a small molecule antagonist of heparan sulfate. Proc. Natl. Acad. Sci. USA.

[26] Aksyuk A.A., Newcomb W.W., Cheng N., Winkler D.C., Fontana J., Heymann J.B., Steven A.C. (2015). Subassemblies and Asymmetry in Assembly of Herpes Simplex Virus Procapsid. Mbio.

[27] Luganini A., Nicoletto S.F., Pizzuto L., Pirri G., Giuliani A., Landolfo S., Gribaudo G. (2011). Inhibition of Herpes Simplex Virus Type 1 and Type 2 Infections by Peptide-Derivatized Dendrimers. Antimicrob. Agents Chemother..

[28] Gescher K., Kühn J., Lorentzen E., Hafezi W., Derksen A., Deters A., Hensel A. (2011). Proanthocyanidin-enriched extract from Myrothamnus flabellifolia Welw. exerts antiviral activity against herpes simplex virus type 1 by inhibition of viral adsorption and penetration. J. Ethnopharmacol..

[29] Donalisio M., Nana H.M., Ngane R.A.N., Gatsing D., Tiabou Tchinda A.T., Rovito R., Cagno V., Cagliero C., Boyom F.F., Rubiolo F. (2013). In Vitro Anti-Herpes Simplex Virus Activity of Crude Extract of the Roots of Nauclea Latifolia Smith (Rubiaceae). BMC Complementary Altern. Med..

[30] Jin F., Zhuo C., He Z., Wang H., Liu W., Zhang R., Wang Y. (2015). Anti-herpes simplex virus activity of polysaccharides from *Eucheuma gelatinae*. World J. Microb. Biot..

[31] Kesharwani A., Polachira S.K., Nair R., Agarwal A., Mishra N.N., Gupta S.K. (2017). Anti-HSV-2 Activity of Terminalia Chebula Retz Extract and Its Constituents, Chebulagic and Chebulinic Acids. BMC Complementary Altern. Med..

[32] Reichling J., Neuner A., Sharaf M., Harkenthl M., Schnitzler P. (2009). Antiviralactivity of Rhus aromatic (fragrant sumac) extracts against two types of hepes simplex viruses in cell culture. Pharmazie.

[33] Lückemeyer D.D., Müller V.D.M., Moritz M.I.G., Stoco P.H., Schenkel E.P., Barardi C.R.M., Reginatto F.H., Simões C.M.O. (2012). Effects of Ilex paraguariensis A.St.Hil.(yerba mate) on herpes simplex virus type 1 and 2 replication. Phytother. Res..

[34] Zhang X., Jia R., Zhou J., Wang M., Yin Z., Cheng A. (2016). Capsid-Targeted Viral Inactivation: A Novel Tactic for Inhibiting Replication in Viral Infections. Viruses.

[35] Guidry J.T., Scott R.S. (2017). The interaction between human papillomavirus and other viruses. Virus Res..

[36] Guan X., Zhang M., Fu M., Luo S., Hu Q. (2019). Herpes simplex virus type 2 immediate early protein ICP27 inhibits IFN-β production in mucosal epithelial cells by antagonizing IRF3 activation. Front. Immunol..

[37] Tamura Y., Nakajima K., Nagayasu K., Takabayashi C. (2002). Flavonoid 5-Glucosides from the Cocoon Shell of the Silkworm, *Bombyx mori*. Phytochemistry.

[38] Bischoff S.C. (2008). Quercetin: Potentials in the prevention and therapy of disease. Curr. Opin. Clin. Nutr. Metab. Care.

[39] Gotoh K., Izumi H., Kanamoto T., Tamada Y., Nakashima H. (2000). Sulfated fibroin, a novel sulfated peptide derived from silk, inhibits human immunodeficiency virus replication in vitro. Biosci. Biotechnol. Biochem..

[40] Jacob J.R., Mansfield K., You J.E., Tennant B.C., Kim Y.H. (2007). Natural Iminosugar Derivatives of 1-Deoxynojirimycin Inhibit Glycosylation of Hepatitis Viral Envelope Proteins. J. Microbiol..

[41] Ponnuvel K.M., Nakazawa H., Furukawa s., Asaoka A., Ishibashi J., Tanaka H., Yamakawa M. (2003). A lipase isolated from the silkworm Bombyx mori shows antiviral activity against nucleopolyhedrovirus. J. Virol..

[42] Zhang J., Liu H., Wei B. (2017). Immune response of T cells during herpes simplex. J. Zhejiang Univ. Sci. B.

[43] Wang H.-Y., Wang Y.-J., Zhou L.-X., Zhuab L., Zhang Y.-Q. (2012). Isolation and Bioactivities of a Non-Sericin Component from Cocoon Shell Silk Sericin of the Silkworm *Bombyx mori*. Food Funct..

[44] Sakudoh T., Sezutsu H., Nakashima T., Kobayashi I., Fujimoto H., Uchino K., Banno Y., Iwano H., Maekawa H., Tamura T. (2007). Carotenoid silk coloration is controlled by a carotenoid-binding protein, a product of the yellow blood gene. Proc. Natl. Acad. Sci. USA.

[45] Kurioka A., Yamazaki M. (2002). Purification and identification of flavonoids from the yellow green cocoon shell (Sasammayu) of the silkworm. Bombyx mori. Biosci. Biotechnol. Biochem..

[46] Yarmolinsky L., Huleihel M., Zaccai M., Ben-Shabat S. (2012). Potent antiviral flavone glycosides from *Ficus benjamina* leaves. Fitoterapia.

[47] Hung P.Y., Ho B.C., Lee S.Y., Chang S.Y., Kao C.L., Lee S.S., Lee C.N. (2015). *Houttuynia cordata* targets the beginning stage of herpes simplex virus infection. PLoS ONE.

[48] Lee S., Lee H.H., Shin Y.S., Kang H., Cho H. (2017). The anti-HSV-1 effect of quercetin is dependent on the suppression of TLR-3 in Raw 264.7 cells. Arch. Pharmacal Res..

[49] Kim C.H., Kim J.E., Song Y.J. (2020). Antiviral activities of quercetin and isoquercitrin against human herpesviruses. Molecules.

