# Supplementary material for: Anti-Herpes Simplex Virus Efficacy of Silk Cocoon, Silkworm Pupa and Non-Sericin Extracts
Source: Antibiotics (Basel). 2021 Dec 19;10(12):1553. doi: 10.3390/antibiotics10121553 (PMC8698825; doi:10.3390/antibiotics10121553)
Supplement: Supplementary file 1 [file antibiotics-10-01553-s001.zip › antibiotics-1475987-supplementary.pdf]

Supplementary Materials

Additional Supplementary Materials may be found in the online version of this article:

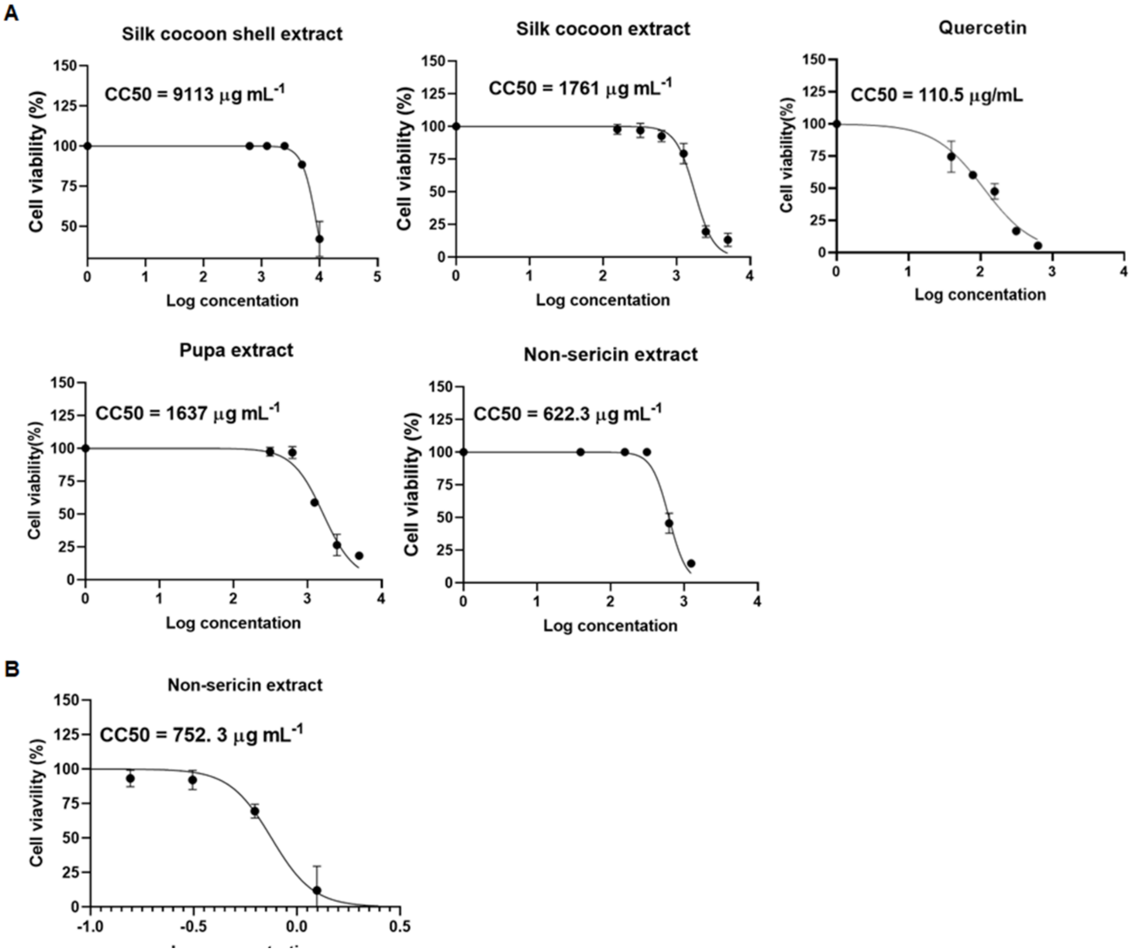

Figure S1: The 50% cytotoxic concentration (CC50) of extracts after treatment on Vero cells (A) with the concentration of silk cocoon shell extract ranging from 625-10,000 µg/mL, silk cocoon extract (156.25 -5,000 µg/mL), silkworm pupa extract (312.50 – 5,000 µg/mL), non-sericin extract (39.06 - 1,250 µg/mL) and quercetin 39.06-625.00 µg/mL. Inaddition, CC50 of non-sericin extract was evaluated on HeLa cell (B) with concentration ranging from 78.12 -2,500 µg/mL.

Table S1. List of primers used to amplify cDNA transcripts levels from HSV-2 infected and non-sericin treated HeLa cell.

Target genes	Forward (5′→3′)	Reverse (5′→3′)
GAPDH	*CGACCACTTTGTCAAGCTCA*	*AGGGGTCTAGGCAACTG*
TNFα	*TGCTTGTTCCTCAGCCTCTT*	*ATGGGCTACAGGCTTGTCACT*
IL8	*TCCTGATTTCTGCAGCTCTGT*	*CCAGACAGAGCTCTCTTCCA*
IL6	*GATCATCCTCGACGGCATC*	*AGCCACTGGTTCTGTGCCT*
IP10	*GAATCGAAGGCCATCAAGAA*	*AAGCAGGGTCAGAACATCCA*
COX-2	*GCAGTTGTTCCAGACAAGCA*	*GAAAGGTGTCAGGCAGAAGG*
